# The menopause transition: a call for a holistic approach

**DOI:** 10.1192/bjb.2025.17

**Published:** 2026-02

**Authors:** Rachel Gibbons

**Affiliations:** Independent clinical researcher, UK

**Keywords:** Menopause, menopause transition, perimenopause, reproductive health, women’s health

## Abstract

This paper advocates for a holistic approach to the menopause transition and challenges the current dominant narrative that frames this transition primarily in biological terms. It examines the psychological, social and cultural dimensions, addresses the stigma faced by older women and advocates for the vital role psychiatrists have to play in supporting postmenopausal women.

‘[There is] a view of menopause as catastrophe […]. If women on the youthful side of the climacteric could glimpse what this state of peaceful potency might be, the difficulty of the transition would be lessened.’

(Germaine Greer^1^)

The Royal College of Psychiatrists established the Menopause Steering Group to address the unmet health needs of women during perimenopause and menopause, following a 2023 Healthcare Safety Investigation Branch (HSIB) report on community mental healthcare in England.^2^

## Personal reflection

At my first Menopause Steering Group meeting a member recounted being asked by a colleague why psychiatrists were involved in discussions about the menopause at all, remarking ‘surely this is the domain of endocrinologists’.

Having recently transitioned through the menopause, this question triggered deep anxiety – a sense that something profoundly complex, central to my experience as a woman, was being reduced to a simple issue of hormones. Although there is considerable variation in women’s experiences of the menopause, I believe many postmenopausal women may share this feeling.^1,3^ Deep down, I know the challenges of the menopause fall squarely within the domain of psychiatry, and I was troubled by the struggle to recognise this vital role.

Reflecting on my own experience, I can attest to the feeling of navigating uncharted territory – rarely communicated effectively. Although undeniably biological, the psychological and social upheaval was profound, affecting every aspect of my identity and relationships. Yet unexpectedly, as Greer and others have described,^1,3,4^ postmenopause has brought me a sense of joy and personal growth, freed from the pressures and expectations that once defined my life.

## Challenging the biological narrative of the menopause

‘Male dominance – and with it the superiority of the male body – was cemented into medicine’s very foundations […] Women were marked by their anatomical difference from men, and medically defined as faulty, defective, deficient.’

(Elinor Cleghorn^5^)

Reducing the menopause transition to biology imposes a male-centric medical model that pathologises a natural life stage. The focus on hormones, although important, overlooks the emotional and social realities women face.

By treating it as a problem to be ‘fixed’, this approach diminishes its complexity, framing it as dysfunction rather than potentially a multifaceted stage of growth.

In his controversial 1966 book *Feminine Forever*,^6^ consultant obstetrician and gynaecologist Robert Wilson encapsulated the stigma and complexities surrounding the menopause. A pioneer in his time, advocating for women’s health in a male-dominated field, Wilson nonetheless framed the menopause as a ‘serious medical condition’ akin to a ‘deficiency disease’ like diabetes, promoting hormone treatments to ‘reverse’ ageing and restore ‘youthfulness’.

His approach implied that women’s value was tied to maintaining sexual vitality and attractiveness, raising concerns about whether the treatment served women or the desires of their male partners. He stated that thanks to hormone prescriptions:

‘The bodily changes of middle age can be reversed, and sexual functions restored […] Instead of witnessing the death of their womanhood, they will remain fully feminine […] The outward signs of this age-defying youthfulness are a straight-backed posture, supple breast contours, taut, smooth skin on face and neck, firm muscle tone, and that particular vigor and grace typical of a healthy female […] At fifty, such women still look attractive in tennis shorts or sleeveless dresses’ (p. 18).^6^

Recent efforts to destigmatise the menopause and deepen the discourse – led by public figures such as Davina McCall, Gwyneth Paltrow and Michelle Obama – have been successful. However, although some recent literature shows increasing awareness of the psychosocial aspects, the focus remains largely biological.

## The nature of the menopause transition

The menopause transition, a natural milestone, typically begins in the person’s 40s. Menopause itself – 12 months without a period – happens at an average age of 51 in Western countries, but symptoms can last years. Just as menarche marks the start of fertility, the menopause signifies its end, bringing the cessation of ovarian function and a decline in hormone levels. These biological changes may be accompanied by physical manifestations, including but not limited to changes in body shape, reduced sexual drive, altered sleep patterns and symptoms such as hot flushes.

The menopause is not merely a biological event but a transition with deep cultural, social and personal significance. In some societies, such as Japan and China, postmenopausal women are revered,^7^ whereas in many Western contexts, older women may face marginalisation and even degradation. For some, the menopause brings freedom, empowerment and relief from issues such as problematic periods or fibroids. For others, it is marked by grief over the loss of fertility, youth and shifts in identity – challenges that can be particularly profound after an early menopause due to medical or biological factors. Significant losses or life trauma can also intensify menopausal symptoms, even decades later.^8^ Therefore acknowledging and grieving these losses is essential, as difficulties in mourning have been linked to mental illness^9^ and may contribute to the increased psychiatric morbidity during this stage.^10^

Nothing illustrates the mind–body connection quite like the menopause. The mind is embodied and the body is en-minded. The menopause reminds us of our inherent transience, with death being the next unavoidable biological event. It becomes the body’s way of challenging the mind’s delusions of immortality, revealing the organic and finite nature of life. Life is short, and the menopause serves as a powerful reminder of this truth.

## The brain–uterus problem

‘In persons of delicate constitution who have inherited a tendency to disease […] the healthy performance of her special functions renders it improbable she will succeed […] For she does not easily regain the vital energy which was recklessly sacrificed in the acquirement of learning’

(Henry Maudsley^11^)

It is well recognised that misogyny has been embedded in medicine throughout much of history.^5^ In retrospect, Henry Maudsley did not cover himself in glory in 1874 when he opposed women entering the medical profession. He supported the popular view of the time that women risked lifelong suffering if they diverted energy from their reproductive systems to intellectual pursuits. This long-standing narrative, pitting the brain against the uterus, has pathologised women’s bodies, framing hormonal changes as signs of weakness. In reality, women’s bodies are incredibly powerful and life-giving. The idea that women are ‘driven mad’ by their biology perpetuates harmful stereotypes, portraying them as erratic, emotional and weak. The current emphasis on biology sustains a reductive view that links hormonal changes to ‘emotional instability’.

## The invisibility of older women: a misconception

‘The changes, the highs and lows and the hormonal shifts, there is power in that. But we were taught to be ashamed of it and to not even seek to understand it or explore it for our own edification, let alone to help the next generation.’

(Michelle Obama^12^)

A key struggle with the menopause is the stigma projected onto older women, making open discussions difficult. Society often unfairly portrays menopausal women as losing their beauty, value and sexuality. The dominant medical model reinforces this stigma by framing menopause as a biological dysfunction, rather than recognising its broader impact on women’s identity, status and role in society. In literature, Greer notes, they are ‘virtually invisible’.^13^ In the media, they are similarly underrepresented and when present, they are frequently portrayed through stereotypes and lack positive role models. In the workplace, they encounter age discrimination and perceptions of decline. Social interactions can lead to dismissal or isolation, and healthcare disparities stem from medical bias and insufficient research. Beauty standards prioritise youth, pressuring older women to conform to cultural expectations of utility, primarily through caregiving.

The term ‘invisible woman syndrome’ discussed in various feminist and sociological studies encapsulates society’s tendency to render older women unseen. However, the idea that women naturally become invisible with age is a societal construct, not a reflection of their true worth or presence. Women are *made* invisible by society, which is entirely different from *becoming* invisible. Why are older women rendered ‘invisible’? One argument is that this invisibility is rooted in societal misogyny and a fear of older women, or ‘anophobia’,^3^ as their newfound postmenopausal power and authenticity are perceived as a threat to the established order:

‘I feel more visible now than I ever did when I was younger. Yes, I was looked at, but only as an object of projection – completely disconnected from who I felt I was inside. Now, I feel I am truly seen. I am more authentic, integrated, and powerful. I care far less about what others think. I am more in touch with my inherent goodness and creativity, which bring me greater joy than at any other time in my life’ (58-year-old psychiatrist, personal conversation).

## Advocacy for a holistic approach to menopause

‘The older woman’s love is not love of herself, nor of herself mirrored in a lover’s eyes, nor is it corrupted by need. It is a feeling of tenderness so still and deep and warm that it gilds every grass blade and blesses every fly. It includes the ones who have a claim on it, and a great deal else besides. I wouldn’t have missed it for the world.’

(Germaine Greer^14^)

In my opinion the anxiety I described in my personal reflection above was – and continues to be – a manifestation of deep anger and frustration, a feeling I believe many older women share in response to being misunderstood and stigmatised. Channelling this anger into advocacy, education and support transforms it into a force for change. As postmenopausal women, we must take responsibility for reshaping the narrative and alleviating the fears younger women may have about this stage of life. And as psychiatrists, we are not just explaining the menopause – we are still working to understand it ourselves. We must approach it with care and curiosity, ensuring we do not contribute to harm. A narrow biological focus reduces our role to prescribers rather than clinicians offering deeper, holistic care.

I hope the Royal College of Psychiatrists will advocate for a balanced, biopsychosocial approach to the menopause – one that challenges hidden stigma and ensures women receive the support they need to navigate this transition. By doing so, we can help society recognise older women not as invisible, but as vibrant, experienced and powerful individuals, and work towards a culture that values and celebrates their contributions at every stage of life.

## Data Availability

Data availability is not applicable to this article as no new data were created or analysed in this study.

## About the author

**Rachel Gibbons** is a consultant psychiatrist, psychoanalyst and group analyst working independently. She is Chair of the Working Group on the Impact of Suicide and Homicide on Clinicians and Vice-Chair of the Psychotherapy Faculty at the Royal College of Psychiatrists, London, UK.
